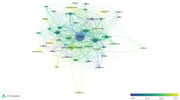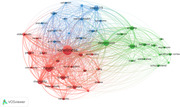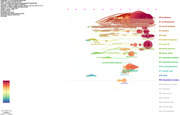# Research Trends on Social Isolation: A Bibliometric Analysis

**DOI:** 10.1002/alz.094801

**Published:** 2025-01-09

**Authors:** HYUN YANG, Jiwon Shin, Sohyeon Yun, Inhye Kim, Hyunseo An, Hae Yean Park

**Affiliations:** ^1^ Yonsei University, Wonju, Wonju Korea, Republic of (South); ^2^ Graduate School, Yonsei University, Wonju, Wonju Korea, Republic of (South); ^3^ Graduate School, Yonsei University, Wonju, Heungup‐meon Korea, Republic of (South); ^4^ College of Software and Digital Healthcare Convergence, Yonsei University, Wonju, Heungup‐meon Korea, Republic of (South)

## Abstract

**Background:**

Research on social isolation has been ongoing, recognizing it as a significant social problem due to the disruption or absence of meaningful relationships providing essential resources for life maintenance. This study aims to analyze the social isolation research trend using quantitative bibliographic methods.

**Method:**

This study collected literature containing 'social isolation' in the title using the Web of Science database and analyzed the number of publications anually and research fields. VOS viewer generated a visualization map based on document relationships, along with academic journals, countries/regions, institutes, and keyword analyses. Additionally, CiteSpace analyzed research keyword trends on social isolation.

**Result:**

From 1980 to March 4, 2024, 2,874 documents were surveyed, with a rapid increase in publications from the 2010s. Neuroscience dominates, with PLOS One leading in publications. The United States leads in research activity, but influential studies from British institutes have a higher total link strength. Keyword analysis highlights ‘Loneliness’, ‘Health’, ‘Depression’, and ‘Mortality’. The top three keyword clusters were classified through the analysis of keywords by time, and each was described as ‘harmfulness of social isolation and loneliness’, ‘social isolation and mental health’ and ‘deepening of social isolation and influencing factors’.

**Conclusion:**

This study identifies research trends in social isolation, providing a basis for future research revival and development